# Size-Controllable Synthesis of Cu Nanoparticles via Hydrogen Reduction at Low Temperatures

**DOI:** 10.3390/ma18102315

**Published:** 2025-05-15

**Authors:** Songya Feng, Anxin Gao, Zihang Ma, Hongjie Xu, Zhiyong Xue

**Affiliations:** Institute for Advanced Materials, North China Electric Power University, Beijing 102206, China

**Keywords:** Cu nanoparticle, high-pressure hydrogen, size-controllable synthesis, polyol method

## Abstract

The general polyol methods used for synthesizing copper nanoparticles (CuNPs) usually require high temperatures to ignite the reduction reaction, which leads to the difficulty of controlling the particle size. Herein, a new polyol method for the size-controllable synthesis of CuNPs at low temperatures by adopting high-pressure hydrogen as the reductant is verified. It is proven that hydrogen is capable to reductively produce CuNPs at temperatures as low as 120 °C. The size of the CuNPs can be controlled between 19.29 nm and 140.46 nm by adjusting the hydrogen pressure, the reaction temperature, and duration. An empirical relationship between temperature and particle size is proposed. This work verifies a low-temperature strategy to synthesize nanoparticles with good size-controllability.

## 1. Introduction

The development of advanced power electronics based on third-generation semiconductors (e.g., SiC, GaN) poses great demand for high-performance electronic packaging materials [1]. Pastes based on Cu nanoparticles (CuNPs) are a group of ideal packaging materials owing to their high surface activity, low sintering temperature, and good electrical/thermal conductivity [2,3,4]. Cu-based pastes composed of CuNPs with optimal size distribution tend to show the closer packing of particles and, thus, better performance [5]. Therefore, CuNPs with controllable sizes are desired.

The synthesis of CuNPs has been extensively studied using physical and chemical approaches. Although the physical methods are advantageous for mass production and cost-effectiveness [6,7,8], they face significant challenges in achieving precise control over particle size distribution and morphological uniformity. Chemical synthesis strategies, including liquid-phase reduction [9,10] and polyol-mediated processes [11], are particularly noteworthy for their ability to regulate nucleation and growth kinetics, allowing for the controlled modulation of particle dimensions. Nonetheless, the liquid-phase reduction method commonly uses toxic inorganic reductants, such as hydrazine hydrate and sodium borohydride, which poses substantial environmental concerns. The polyol method offers the advantage of low toxicity, with polyols serving both as solvents and reductants in the reaction system [12]. Therefore, the polyol method has the potential for industrial-scale fabrication.

Polyols, when compared to inorganic reductants, often exhibit weaker reducibility and slower reaction rates. To overcome these limitations, the most efficient strategies include elevating the reaction temperature and increasing the pH of the system [13,14]. However, raising the reaction temperature or pH accelerates the growth kinetics of the Cu nuclei, leading to the rapid growth of the CuNPs, which brings about the difficulty of controlling the size. For this concern, an improved polyol method operating at low-reaction temperatures needs to be proposed to fabricate size-controllable CuNPs.

In this work, the size-controllable synthesis of CuNPs was achieved at low temperatures by adopting high-pressure hydrogen as the reductant in a polyol (ethylene glycol) system. CuNPs can be fabricated at temperatures as low as 120 °C in the reaction system. The reduction effect of hydrogen was investigated. The size dependence of CuNPs on hydrogen pressure, the reaction temperature, and duration was studied using X-ray diffraction (XRD) and transmission electron microscopy (TEM). This work verifies a low-temperature polyol strategy to synthesize CuNPs with good size-controllability.

## 2. Materials and Methods

### 2.1. Materials

Copper chloride (CuCl_2_, AR) was purchased from Shanghai Aladdin Biochemical Technology Co., Ltd. (Shanghai, China); sodium hydroxide (NaOH, AR) and anhydrous ethanol (AR) were purchased from Sinopharm (Shanghai, China); and ethylene glycol (EG, AR) and polyvinylpyrrolidone (PVP, K29-32) were purchased from Shanghai Macklin Biochemical Co., Ltd. (Shanghai, China).

### 2.2. Preparation and Characterization of CuNPs

PVP was first dissolved in EG at 70 °C to produce a 20 wt.% solution; 2.9 mmol of CuCl_2_ was then dispersed in 50 mL of the solution to obtain solution A. In total, 14.7 mL of EG dissolved with 0.5 mol/L NaOH was added dropwise to solution A, which yielded the blue colloid B. Colloid B was transferred into a high-pressure reactor for producing CuNPs at various conditions, i.e., temperatures from 120 to 220 °C, H_2_ pressure from 1.0 to 1.8 MPa, and reaction durations from 0.5 to 1.5 h. The selection of the condition was decided by referring to previous studies. Upon cooling the reactor to room temperature, the products were separated by centrifuging at 10,000 rpm for 15 min. The obtained solids were washed at least three times with anhydrous ethanol and deionized water alternately, followed by drying in a vacuum freeze-dryer for 12 h, and they were finally milled to obtain CuNPs.

The microstructure of the CuNPs was characterized by a Talos F200X field emission transmission electron microscope (TEM, Thermo Scientific, Waltham, MA, USA). The diameters of 100 particles were measured to give the average size and the standard deviation by adopting the normal distribution model. The crystal structure of the particles was characterized by DX2700BH X-ray diffraction (XRD, Hao Yuan, Dandong, China).

## 3. Results and Discussion

### 3.1. Verification of H_2_ as the Reductant

**Figure 1:**
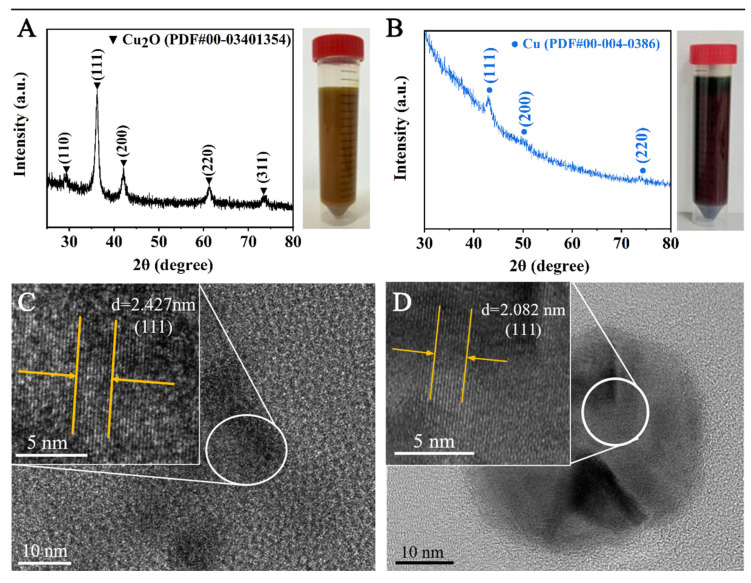
XRD patterns of the obtained products by reacting at 130 °C for 0.5 h in (**A**) N_2_ and (**B**) H_2_ atmospheres of 1.6 MPa. The pictures show the color of the products. Corresponding TEM images of the products synthesized are shown in (**C**) N_2_ and (**D**) H_2_.

**Figure 2:**
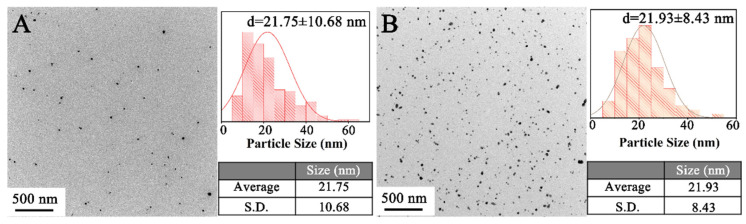
TEM images and results of the size statistics of the products obtained in (**A**) H_2_ for 0.5 h and (**B**) H_2_ for 0.5 h, followed by N_2_ for 1 h.

Blue colloid B was subject to a reaction at 130 °C for 0.5 h in the atmosphere of 1.6 MPa H_2_ or N_2_. As shown in Figure 1A, an XRD investigation is conducted to verify the phase constitution of the product obtained from reacting in N_2_. A series of (111) and (200) crystal planes of Cu_2_O are identified at 2θ = 36.25 and 41.97, implying the formation of Cu_2_O [15]. TEM was used to further characterize the microstructure. As displayed in Figure 1C, the obtained nanocrystalline shows a plane spacing of 0.243 nm, which corresponds to the (111) crystal plane of Cu_2_O [16]. Such results suggest that EG can only reduce Cu^2+^ to Cu^+^, rather than Cu, due to its insufficient reducibility at the relatively low temperature of 130 °C. In contrast, the XRD pattern shows a set of peaks characteristic of face-centered cubic Cu for the product obtained in the H_2_ atmosphere, which is further verified by the typical (111) crystal plane of Cu with a plane spacing of 0.208 nm in the TEM micrograph (Figure 1D) [17]. The average particle size is measured to be 21.75 ± 10.68 nm (Figure 2A). Therefore, H_2_ plays a key role in reducing the Cu^2+^ precursor to Cu at the condition of 130 °C.

**Figure 3:**
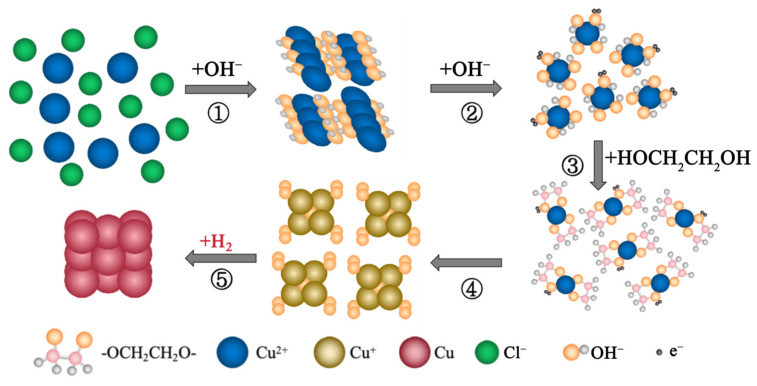
Schematic illustration of the forming processes of the CuNPs in H_2_ atmospheres at low temperatures. Step1: Cu^2+^ reacts with OH^−^ to form the Cu(OH)_2_ precipitate; Step 2: OH^−^ further reacts with Cu(OH)_2_ to form complex [Cu(OH)_4_]^2−^; Step 3: [Cu(OH)_4_]^2−^ then reacts with EG to form [Cu(OCH_2_CH_2_O)_2_]^2−^; Step 4: [Cu(OCH_2_CH_2_O)_2_]^2−^ decomposes into Cu_2_O upon heating; Step 5: Cu_2_O is reduced to metallic Cu by H_2_ at the adopted temperature.

To further verify whether H_2_ acted as the reductant to obtain CuNPs at 130 °C, a two-step experiment was conducted by replacing the H_2_ atmosphere with N_2_ halfway through the reaction. Initially, the reactor was filled with H_2_ at 1.6 MPa and proceeded at 130 °C for 0.5 h. In the second step, H_2_ was replaced by N_2_, and the process continued for an additional hour. The size of the generated particles is statistically measured by referring to the TEM image (Figure 2B). The average size is estimated to be 21.93 ± 8.43 nm, which is almost the same compared to the value of 21.75 ± 10.68 nm obtained without further reaction in N_2_. Such phenomena demonstrate that the reduction reaction ceases after expelling H_2_, confirming that the formation of CuNPs is attributed to reductive H_2_ at the temperature of 130 °C. The reaction mechanism is schematically illustrated in Figure 3. With the addition of alkaline, Cu^2+^ initially reacts with OH^−^ to form the Cu(OH)_2_ precipitate and the complex [Cu(OH)_4_]^2−^, as described by Steps 1 and 2. Subsequently, [Cu(OH)_4_]^2−^ further reacts with EG and forms [Cu(OCH_2_CH_2_O)_2_]^2−^, as shown in Step 3. Upon heating, this complex decomposes into Cu_2_O, as depicted in Step 4 [18]. Finally, Cu_2_O is reduced to metallic Cu by H_2_ at the adopted temperature (Step 5). The chemical equations for each step are shown as Equations (1)–(5):

Cu^2+^ + 2OH^−^ = Cu(OH)_2_ ↓ (1)

Cu(OH)_2_ + 2OH^−^ = [Cu(OH)_4_]^2−^ (2)

[Cu(OH)_4_]^2−^ + 2HOCH_2_CH_2_OH = [Cu(OCH_2_CH_2_O)_2_]^2−^ + 4H_2_O (3)

2[Cu(OCH_2_CH_2_O)_2_]^2−^ = CH_3_COCOCH_3_ + 2(OCH_2_CH_2_O)^2−^ + H_2_O + Cu_2_O (4)

Cu_2_O + H_2_ = 2Cu + H_2_O (5)

### 3.2. Control of the CuNP Size

#### 3.2.1. H_2_ Pressure

**Figure 4:**
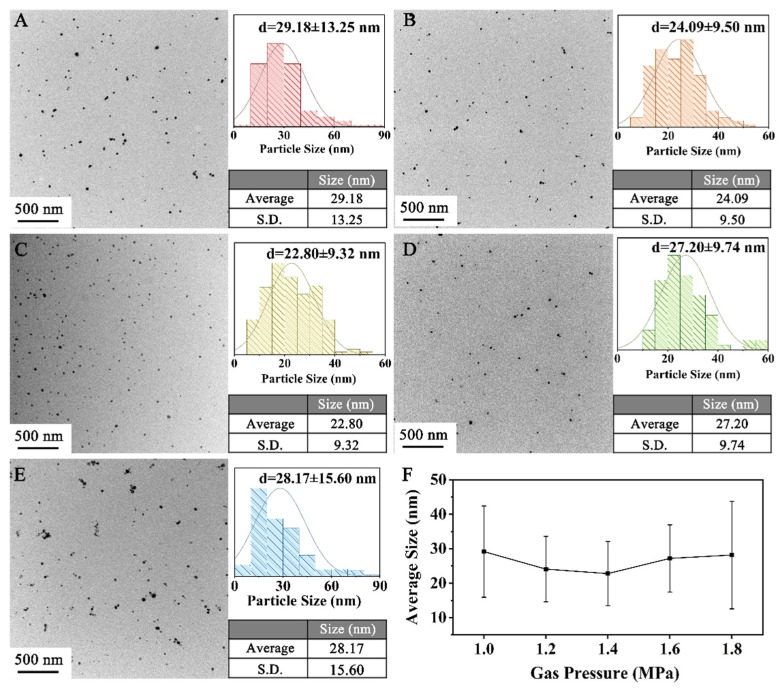
Particle size of products prepared under different pressures: (**A**) 1.0 MPa, (**B**) 1.2 MPa, (**C**) 1.4 MPa, (**D**) 1.6 MPa and (**E**) 1.8 MPa. (**F**) The relationship between reaction pressures and CuNP size.

H_2_ is used as the reductant in the reaction, and the Cu_2_O species, decomposed from the complex [Cu(OCH_2_CH_2_O)_2_]^2−^, is reduced to metallic Cu. Since the solubility of H_2_ in the EG solution is proportional to the pressure of gaseous H_2_ according to Henry’s law [19], the concentration of the dissolved H_2_ is adjusted by applying H_2_ pressure in the range of 1.0–1.8 MPa. The reactions run at 130 °C for 0.5 h. The sizes of the CuNPs synthesized at different pressures were characterized, and the results are shown in Figure 4. As summarized in Figure 4F, the size first decreases from 29.18 ± 13.25 nm for 1.0 MPa to 22.80 ± 9.32 nm for 1.4 MPa and then increases to 28.17 ± 15.60 for 1.8 MPa. The size of the CuNPs shows a V-shaped trend with pressure, probably owing to the switch of nucleation-dominant processes to growth-dominant ones. At low H_2_ pressures (1.0~1.2 MPa), the nucleation processes govern the formation of the CuNPs. Few H_2_ molecules are dissolved in the EG solution, which leads to a low nuclei rate. Owing to the abundant supply of Cu^2+^, a few Cu nuclei undergo free growth, which results in large CuNPs. As the H_2_ pressure rises, the number of Cu nuclei increases, leading to an intensified competition for Cu^2+^ supply. As a result, Cu^2+^ is insufficient to support the free growth of these particles, which causes a decrease in the CuNP size. At higher pressures of above 1.4 MPa, the growth processes become dominant. Higher H_2_ pressure increases the concentration of reductant H_2_ in the EG solution, thus increasing the reduction potential. Therefore, the growth processes of CuNPs are promoted, which finally leads to a larger size.

#### 3.2.2. Reaction Temperature

**Figure 5:**
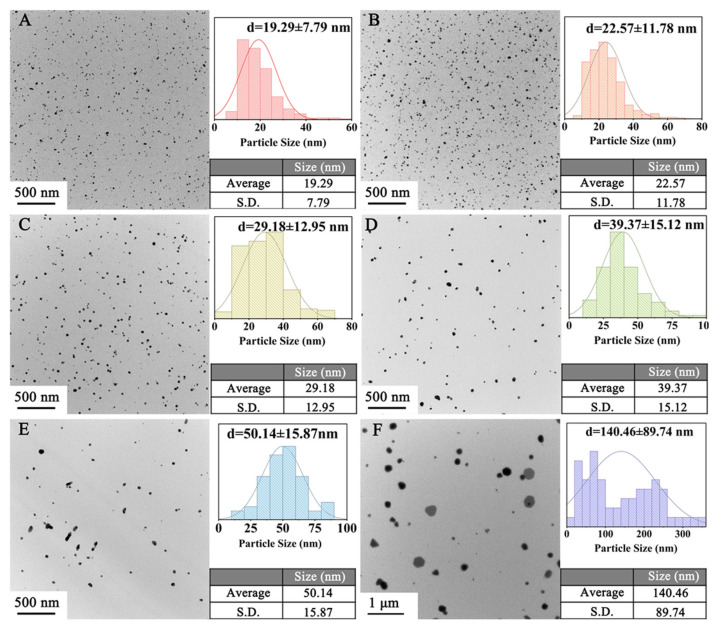
Particle size of products prepared under different temperatures: (**A**) 120 °C, (**B**) 130 °C, (**C**) 140 °C, (**D**) 150 °C, (**E**) 160 °C, and (**F**) 220 °C.

**Figure 6:**
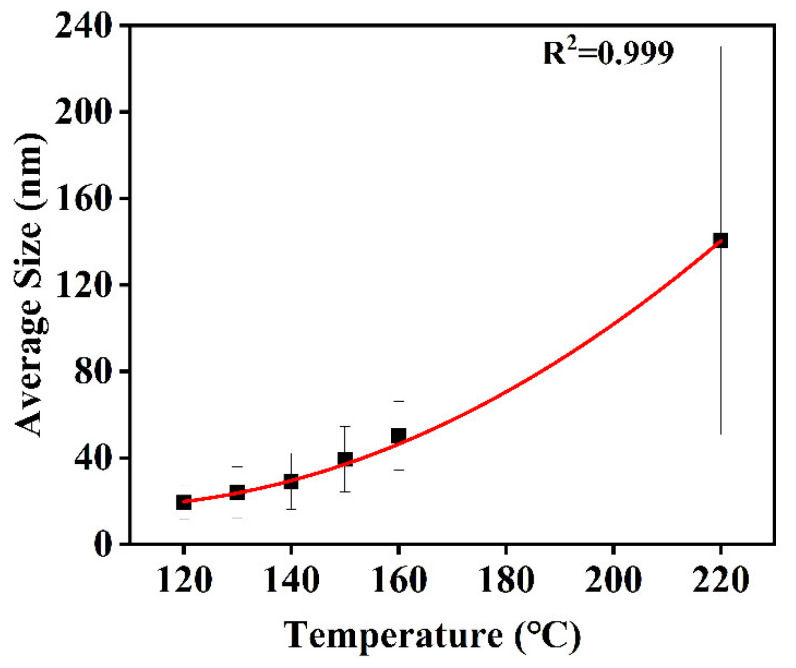
The relationship between the reaction temperature and size of the CuNPs. The red curve is the empirical fitting of the plots.

**Figure 7:**
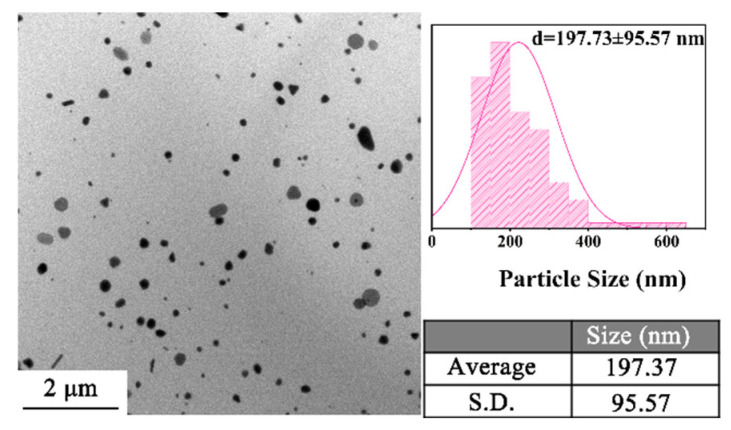
TEM image and particle size of the products prepared at 220 °C for 0.5 h in a N_2_ atmosphere.

Temperature is a key factor affecting particle size. The temperature range of 120–220 °C was chosen to explore the effect of temperature on the CuNP size under the H_2_ pressure of 1.4 MPa for 0.5 h. TEM results suggest that the sizes of the CuNPs synthesized vary with temperature, as displayed in Figure 5. As shown in Figure 5A, the low temperature of 120 °C can generate CuNPs with an ultrafine size of 19.29 ± 7.79 nm, which suggests that the nucleation process can be activated at this temperature. With the increase in the reacting temperature, the reductivity of H_2_ is stronger, which leads to the speeding up of the conversion from Cu^2+^ to Cu. The growth rate of the CuNPs thus increases. Therefore, the sizes of the CuNPs present an increasing trend with temperature, as summarized in Figure 6. The size of the CuNPs can be empirically fitted by the relationship of d = 0.00902 (T−103.104)^2^ + 17.345 (R^2^ = 0.999), where d is the diameter of the CuNPs and T is the temperature. The squared relationship between d and T indicates that the growth rate of the particles becomes faster at high temperatures, which is particularly obvious at 220 °C. To reveal the underlying mechanism, a supplementary experiment was conducted at 220 °C for 0.5 h in a N_2_ atmosphere. The results show that CuNPs are obtained with a size of 197.37 ± 95.57 nm (Figure 7), which indicates that EG is capable of acting as the reductant at 220 °C, consistent with the reported work [20]. Therefore, the squared relationship is probably ascribed to the involvement of EG as the secondary reductant at high temperatures.

#### 3.2.3. Reaction Duration

**Figure 8:**
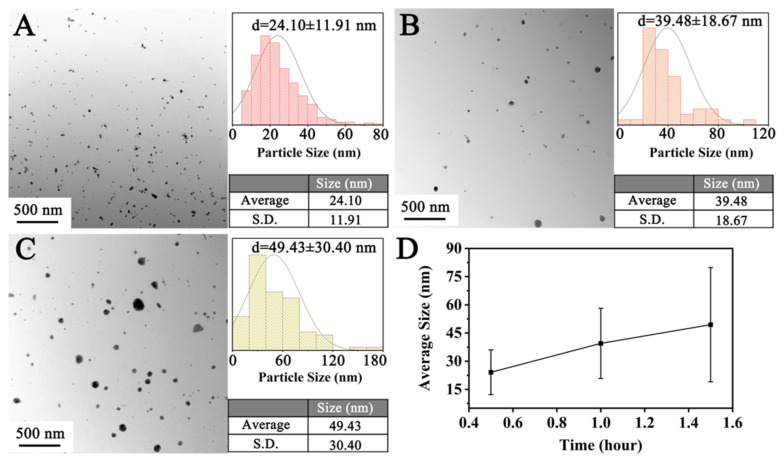
Particle size of products prepared for durations of (**A**) 0.5 h, (**B**) 1 h, and (**C**) 1.5 h. (**D**) The relationship between the reaction time and size of the CuNPs.

The size of the CuNPs is also related to the reaction duration, as shown in Figure 8. At a low temperature of 130 °C, the kinetics for both diffusion and reduction are relatively slow, which leads to the gradual growth of the CuNP size within the reaction duration. The average size of the CuNPs increases slightly from 24.10 ± 11.91 nm to 39.48 ± 18.67 nm when the reaction duration extends from 0.5 h to 1 h. Further elongation of the duration to 1.5 h only results in a particle size of 49.43 ± 30.40 nm. The insensitivity of the particle size with duration implies a large operation window to control the size of the CuNPs.

### 3.3. Outlook of the CuNPs

By adopting H_2_ as the reductant in a polyol system, the reaction temperature to obtain CuNPs is reduced to 120 °C, which is significantly lower than the reaction in EG. The size of the CuNPs increases with temperature and duration, which is consistent with reported works [15,21]. As for H_2_, the present work showed an unexpected V-shaped trend in CuNP size with H_2_ pressure, which is distinct from typical synthesis methods. However, this method still suffers from insufficient production efficiency due to the low concentration of reactants, which should be further optimized in subsequent studies. The present CuNPs are expected to be used as the conductive phase to fabricate packaging pastes for the high-density interconnection of advanced power electronics. Commonly, a high packing density is desired for the conductive phase to optimize the connection. For conductive phases that consist of, for example, two kinds of particles with different sizes, the packing density of the conductive phase relies on the relative size of the two particles. The present size-controllable CuNPs enable optimal size matching between two kinds of Cu particles with different sizes. Therefore, the more densely packed the Cu particles, the higher the performance connection of the power electronics.

## 4. Conclusions

A new method has been developed for the size-controllable preparation of CuNPs using EG as the solvent and H_2_ as the reductant. The reduction effect of H_2_ in producing CuNPs is proven by replacing H_2_ with N_2_ halfway through the reaction. The particle size can be controlled by adjusting the H_2_ pressure, reaction temperature, and time. The particle size shows a V-shaped trend in the range of 1.0 MPa to 1.8 MPa, reaching a minimum of 22.80 ± 9.32 nm at 1.4 MPa, probably owing to the switch from nucleation-dominant processes to growth-dominant ones. The size of the CuNPs increases from 19.29 ± 7.79 nm to 140.46 ± 89.74 nm with the temperature increasing from 120 °C to 220 °C and from 24.10 ± 11.91 nm to 49.43 ± 30.40 nm with the reaction time extending from 0.5 h to 1.5 h. An empirical relationship between temperature (T) and particle size (d) is established, expressed as d = 0.00902 (T − 103.104)^2^ + 17.345. This work proposes a low-temperature strategy to synthesize CuNPs with controllable sizes and verifies H_2_ as a potential regent to fabricate nanoparticles in a moderate way.

## Data Availability

The original contributions presented in this study are included in the article. Further inquiries can be directed to the corresponding author.

## Abbreviations

The following abbreviations are used in this manuscript:

CuNPs	Copper nanoparticles
EG	Ethylene glycol
PVP	Polyvinylpyrrolidone
XRD	X-ray diffraction
TEM	Transmission electron microscope
S.D.	Standard deviation
